# Chung–Jansen Syndrome in a Young Woman with a PHIP Variant: Severe Obesity, Intellectual Disability, and Endocrine Abnormalities

**DOI:** 10.3390/jcm15124609

**Published:** 2026-06-13

**Authors:** Francesco Donno, Federica Bianco, Roberta Schininà, Rita Selvatici, Giuseppina Stoico, Alessandra Ferlini, Alberto Gobbo, Maria Chiara Zatelli, Stefania Bigoni, Maria Rosaria Ambrosio

**Affiliations:** 1 Section of Endocrinology, Geriatrics and Internal Medicine, Department of Medical Sciences, University of Ferrara, 44124 Ferrara, Italy; francesco.donno@edu.unife.it (F.D.); federica.bianco@edu.unife.it (F.B.); alberto.gobbo@edu.unife.it (A.G.); ztlmch@unife.it (M.C.Z.); 2 Medical Genetics Unit, Department of Medical Science and Department of Mother and Child, Ferrara University and Ferrara University Hospital, 44124 Ferrara, Italy; roberta.schinina@unife.it (R.S.); rita.selvatici@unife.it (R.S.); giuseppina.stoico@ospfe.it (G.S.); fla@unife.it (A.F.); stefania.bigoni@unife.it (S.B.); 3 Endocrine Unit, University Hospital S. Anna, 44124 Ferrara, Italy

**Keywords:** facial dysmorphisms, behavioural disturbances, endocrine dysfunctions, case report, next generation sequencing

## Abstract

**Background:** Chung–Jansen syndrome (CHUJANS) is a rare autosomal dominant genetic condition caused by pathogenic variants in the *PHIP* gene, which encodes a protein involved in neurodevelopmental processes and IGF-1 signalling. The phenotype is characterised by variable degrees of intellectual disability, early-onset obesity or overweight, distinctive facial dysmorphisms, and behavioural disturbances. We here present a case of Chung–Jansen syndrome with a detailed endocrine work-up, highlighting the metabolic component of this syndrome. **Case Presentation:** We describe the case of a 21-year-old woman referred to our centre for evaluation of oligomenorrhea in the context of severe obesity (BMI 50.4 kg/m^2^), short stature (151 cm, <3rd percentile), and moderate-to-severe intellectual disability (full-scale IQ 38). Physical examination revealed dysmorphic features, including a round face, upslanting palpebral fissures, prominent zygomatic bones, anteverted nares, a prominent chin, and bilateral brachydactyly type E1. Laboratory investigations documented subclinical primary hypothyroidism of autoimmune origin, impaired glucose tolerance with associated hyperinsulinism, and polyendocrine metabolic ovarian syndrome (PMOS, previously known as PCOS). Exome analysis by next-generation sequencing (NGS) identified a heterozygous c.328C>T [p.(Arg110Cys)] variant in the *PHIP* gene, already reported in literature and classified as likely pathogenic (ACMG class 4). Segregation analysis in the mother (father was not available for the test) did not reveal the variant, suggesting a de novo origin in the patient. Concurrently, the same analysis revealed a variant of uncertain significance in the *ANKRD17* gene, while array-CGH detected a maternally inherited microdeletion of uncertain significance on chromosome X (Xp11.23). **Conclusions:** This case confirms the association between the *PHIP* p.(Arg110Cys) variant and the phenotype of Chung–Jansen syndrome, providing a detailed characterisation of the endocrine and psychiatric comorbidities. Indeed, our report expands the knowledge on the endocrine phenotype providing further suggestion for personalised patient management. It underscores the importance of NGS in the diagnostic workup of syndromic obesity with intellectual disability, especially in the presence of negative family history and prior inconclusive genetic testing. This case suggests the inclusion of comprehensive endocrine evaluations in future studies on patients with Chung–Jansen syndrome, in order to support endocrine work-up and facilitate early identification and appropriate management of potentially treatable alterations.

## 1. Introduction

Chung–Jansen syndrome (CHUJANS; OMIM #617991) is a rare autosomal dominant genetic condition, first described in 2016 [1], caused by pathogenic or likely pathogenic variants in the *PHIP* (pleckstrin homology domain interacting protein) gene, located on chromosome 6q14.1 [1,2]. The gene encodes two protein isoforms, DCAF14 and NDRP, both implicated in fundamental neurodevelopmental processes, including gene transcription regulation and IGF-1 signalling, playing a key role in neuronal growth and energy metabolism [3].

The two isoforms exhibit distinct cellular localisations. The cytoplasmic isoform (104 kDa) interacts with insulin receptor substrate (IRS)-1 and -2 and is required for insulin and insulin-like growth factor (IGF-1) signalling. The nuclear isoform (230 kDa) is localised exclusively in the nucleus. Nuclear PHIP (DDB1- and CUL4-associated factor 14 [DCAF14] or replication initiation determinant protein [REPID]) is known to bind directly to chromatin to promote DNA replication initiation and gene transcription. In the nucleus, wild-type PHIP protein directly potentiates the transcription of proopiomelanocortin (*POMC*), a neuropeptide fundamental for appetite suppression. Normal PHIP activity thus contributes to maintaining energy homeostasis and satiety. Pathogenic *PHIP* variants repress *POMC* transcription, causing reduced expression and consequently promoting the development of obesity [4].

The phenotypic spectrum of the syndrome is broad and variable, even within the same family [5,6]. The main features include intellectual disability of variable severity, ranging from mild to moderate or, more rarely, severe; early-onset obesity or overweight, often with onset in infancy or adolescence, frequently associated with hyperphagia and eating behaviour disorders; characteristic dysmorphic features, such as a high forehead, prominent supraorbital ridges, thick eyebrows, upslanting palpebral fissures, prominent zygomatic bones, anteverted nares, a thin upper lip, a long philtrum, and large ears with prominent lobes [2,5]. To these are added behavioural disorders such as impulsivity, aggression, motor hyperactivity, anxiety, and autism spectrum traits, as well as minor neurological abnormalities such as hypotonia and balance disorders, skeletal abnormalities such as brachydactyly, and ophthalmological (myopia, hyperopia, strabismus) and gastrointestinal (constipation) issues [5,6].

The clinical picture raises diagnostic suspicion, which is confirmed by genetic evaluation based on the identification of pathogenic *PHIP* variants using next-generation sequencing (NGS) techniques, often within multigene panels for intellectual disability or syndromic obesity, or through Whole Exome Sequencing (WES). Inheritance is autosomal dominant, and most variants described in the literature have arisen de novo, although cases of familial inheritance with variable penetrance have been reported [6].

We describe the case of a young woman with a classic phenotype of Chung–Jansen syndrome, in whom NGS identified the likely pathogenic variant c.328C>T [p.(Arg110Cys)] in the *PHIP* gene, not inherited from the mother and therefore presumably de novo. Furthermore, we performed a comprehensive investigation of the endocrine phenotype, characterised not only by obesity, shared by a substantial proportion of affected individuals, but also by other endocrine aspects of the phenotype.

This case offers the opportunity for a detailed clinical, endocrinological, and psychiatric characterisation of the syndrome, contributing to defining the phenotypic spectrum and underscoring the importance of a multidisciplinary approach in the management of these patients, especially concerning the endocrine aspects that may be neglected and remain insufficiently characterised.

## 2. Case Presentation

### 2.1. Patient Information

A 21-year-old woman of Caucasian ethnicity, third-born daughter of non-consanguineous parents, was referred to our centre for evaluation of oligomenorrhea in the context of severe obesity and intellectual disability. Written informed consent was obtained for the publication of the clinical case and related images, in accordance with the ethical standards of the Institutional Committee.

She was born from an uneventful pregnancy, by caesarean section, due to a previous caesarean section. Fetal karyotype analysis on amniotic fluid, performed due to maternal age (35 years), was normal. During the first year of life, hypotonia and relative macrocephaly were observed in absence of sucking/swallowing problems. Psychomotor development was delayed: sitting at ~1-year, independent ambulation between 2–3 years and language with greater delay (first words > 2 years, complex sentences only after 4–5 years). Some genetic investigations had been performed in the past, including molecular analysis for Borjeson–Forssman–Lehmann syndrome, but all are reported as negative.

The patient resided in an educational community for minors from age 12 to 20 and then went back living with her family, participating actively in social interactions.

Menarche occurred at 14 years of age, followed by progressively worsening oligomenorrhea leading to secondary amenorrhea. Home therapy at the time of evaluation included lithium carbonate for the management of behavioural disturbances.

The family history reports the presence of obesity in the mother, aged 56 years, and in her family in the absence of further elements of increased genetic risk. The father is reported to be in good health. Despite the lack of direct information about his family, his two brothers are reported to be in good health.

The patient has a 25-year-old brother diagnosed with type 1 diabetes mellitus and dysgraphia, and a 23-year-old sister who is in good health.

### 2.2. Clinical Findings and Diagnostic Assessments

On physical examination, the patient was alert and cooperative, with fluent speech characterised by simple sentences and the use of “passe-partout” words. Motor tics and manual stereotypies were observed. Auxological parameters documented a height of 151 cm, below the third percentile for age and sex, with an arm span of 161.5 cm. Weight was 115 kg (well above the 97th percentile) with a waist circumference of 159.5 cm, also above the 95th percentile. Calculated BMI was 50.4 kg/m^2^, indicating class III obesity, while the WHtR was 1.06.

**Figure 1:**
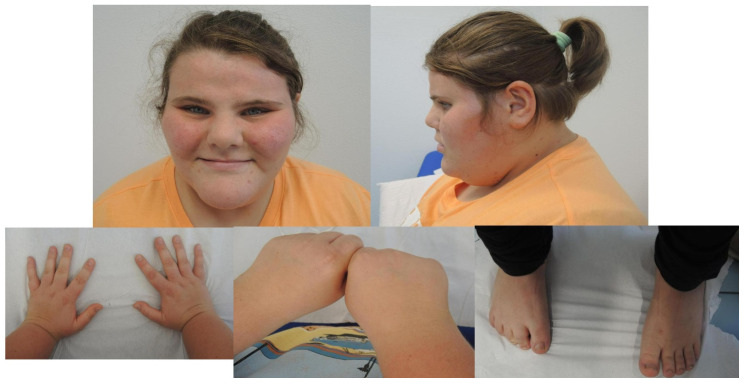
Dysmorphisms of our patient.

**Figure 2:**
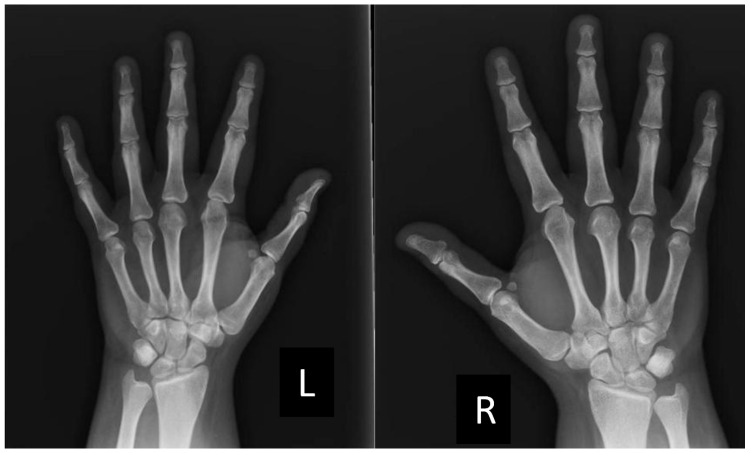
Bilateral brachydactyly of the fourth metacarpals. L = left hand, R = right hand.

Dysmorphology examination revealed facial dysmorphic features including triangular facies, upslanted palpebral fissures, prominent zygomatic bones, anteverted nares, prominent chin, thin upper lip vermilion, and mandibular prognathia (Figure 1). Apparent brachydactyly (IV ray of both hands), a feature of Chung–Jansen syndrome, was subsequently confirmed by radiographic examination for the fourth metacarpal bilaterally (Figure 2). Low-pitched voice was also observed.

#### 2.2.1. Psychiatric Findings

Psychiatric evaluation showed anxiety attacks and past anger episodes managed with self-regulation strategies learned in the community. No psychosis or self-harm ideation was present. The patient described occasional judgmental “inner voices” during stress or fatigue, which she can dismiss using cognitive strategies, though these are less effective when alone and work only with staff support. Mental status examination revealed a lucid, oriented, cooperative patient with congruent mood. No thought disorders or heteroaggressive risk were identified. Based on the clinical picture, it was recommended to add quetiapine 50 mg/day as needed to manage acute anxiety.

#### 2.2.2. Neurologic Findings

Neuropsychological evaluation using the WAIS-IV scale revealed a globally deficient profile (Table 1). During the examination, the patient was alert and cooperative, well-oriented to personal details, with fluent speech albeit characterised by simple sentences and the use of passe-partout words. Mild performance anxiety with psychomotor agitation emerged, manifested through stereotypies and environmental dependency, which tended to increase as the evaluation progressed.

Results were globally deficient in all test sections, without preserved islands of ability, with values at the lower limits of the scale in all subtests. This profile indicates a moderate-to-severe intellectual deficit. No epileptic seizures were reported or documented at any stage of life. Neurological examination revealed occasional stereotyped hand movements, with preserved muscle tone and symmetrical deep tendon reflexes.

#### 2.2.3. Endocrinologic Findings

From the endocrinological point of view, laboratory tests documented subclinical primary hypothyroidism of autoimmune origin, with TSH 6.8 µIU/mL (normal range 0.4–4.0), FT3 and FT4 at the lower limits of normal, and positivity for anti-thyroperoxidase and anti-thyroglobulin antibodies. Glucose metabolism was impaired, with fasting glucose 98 mg/dL, insulin 28 µIU/mL (reference value < 15 µIU/mL), and HOMA-IR 6.8 (cut-off for normal insulin sensitivity < 2.5), indicative of hyperinsulinism with reduced insulin sensitivity, suggesting a picture of severe insulin resistance. Oral glucose tolerance test confirmed impaired glucose tolerance, with a 120 min plasma glucose concentration of 145 mg/dL (normal value < 140 mg/dL) and a peak insulin level of 119.4 µIU/mL. HbA1c was within the normal range (31 mmol/mol). Cortisol levels were normally suppressed after Nugent testing.

Our patient presented with polyendocrine metabolic ovarian syndrome (PMOS, previously known as PCOS). Laboratory tests showed total testosterone levels of 0.65 ng/mL and free testosterone of 14.40 pg/mL; abdominal ultrasound showed the presence of follicular microcysts at the ovarian level.

#### 2.2.4. Nephrologic and Cardiologic Findings

Additionally, mild grade I hydronephrosis on the right side, likely functional, was documented. Electrocardiogram was within normal limits.

#### 2.2.5. Ophthalmologic Findings

Ophthalmological evaluation revealed a refractive error corrected with lenses, with visual acuity of 10/10 in both eyes after correction and normal fundus oculi.

#### 2.2.6. Genetic Findings

Chromosomal Microarray Analysis with CytoChip genomic array ISCA v2 OGT 8x60 (GRCh37/hg19) identified a maternal 5 kb microdeletion of uncertain significance, being described in reference databases in both healthy individuals and patients with neurodevelopmental disorders, involving the short arm of X chromosome, introducing a breakpoint in OMIM Morbid ZNF41 gene (*314995) (arr[hg19]Xp11.23(47330209_47334976)x1 mat).

Subsequently, Whole Exome Sequencing (WES) was performed by using Illumina DNA Prep with Exome 2.5 Enrichment for target region capture. Qualified captured library was loaded on the NextSeq550DX Illumina platform using 150 bp paired-end reads. Raw data were analysed using Emedgene software version 37, including alignment, variant calling, copy number variation (CNV) analysis, and variant annotation. Sequencing reads were aligned to the human genome build GRCh37/UCSC hg19. The average target coverage was 161.2X, with ≥20X coverage achieved for 98% of target regions. The identified variants were confirmed with Sanger Sequencing.

Targeted analysis of genes associated with the patient’s clinical features, defined according to HPO terminology, identified a heterozygous missense variant in exon 5 of the *PHIP* gene NM_017934.7: c.328C>T; p.(Arg110Cys), classified as likely pathogenic (ACMG Class 4) based on ACMG/AMP criteria (PS2, PM2, PM5, PP2). Pathogenic variants in *PHIP* are associated with Chung–Jansen syndrome (Table 2).

The identified variant results in the substitution of arginine with cysteine at codon 110. This residue is evolutionarily conserved, and missense variation at this position is considered functionally relevant. The variant is reported in the ClinVar database (ID: 975951), is absent from large population databases (e.g., gnomAD), and has been previously described in a limited number of individuals with Chung–Jansen syndrome, in whom it occurred de novo.

In silico analysis of protein sequence and biophysical properties, including structural and functional features, amino acid conservation, physicochemical variation, residue mobility, and thermodynamic stability, was performed using AlphaMissense [7]. The identified PHIP missense variant was predicted to have a deleterious effect on protein function, potentially consistent with a loss-of-function mechanism. The variant achieved a high AlphaMissense pathogenicity score (0.971), corresponding to the model’s high-confidence threshold and an estimated positive predictive value of 95% as calibrated against ClinVar data.

**Figure 3:**
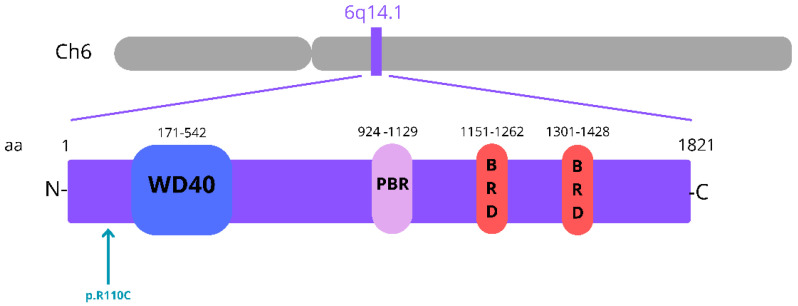
The PHIP gene (NM_017934.6), located on the long arm of chromosome 6 in the 6q14.1 region, encodes a 1821 amino acids long isoform, PHIP/DCAF14. The shorter isoform, NDRP, consists in a 1019 amino acids protein. The full-length protein consists of four functional domains: WD40 (amino acids 171–542), PBR (amino acids 924–1129) and BRD domains (amino acids 1151–1262 and amino acids 1301–1428). In light blue, the mutation identified in our patient.

The *PHIP* gene, located on chromosome 6q14.1, encodes protein isoforms involved in neurodevelopmental processes, including regulation of pro-opiomelanocortin (POMC) expression, a precursor involved in satiety and energy homeostasis (Figure 3).

The same analysis identified an additional heterozygous missense variant in exon 20 of the *ANKRD17* gene (NM_032217.5: c.3755G>A; p.(Arg1252Gln)). This variant is absent from population databases (MAF = 0 in gnomAD), has not been reported in the literature, and is currently classified in the ClinVar database (ID: 2574502) as a variant of uncertain significance (VUS; ACMG Class 3).

The *ANKRD17* gene is associated with Chopra–Amiel–Gordon syndrome (CAGS), an autosomal dominant disorder characterised by neurodevelopmental delay with intellectual disability, language impairment, growth retardation, facial dysmorphisms, feeding difficulties, ophthalmological abnormalities, balance disturbances, and susceptibility to recurrent infections.

Given the current classification of the variant and the lack of significant phenotypic overlap with the patient’s clinical presentation, this variant is not considered causative of the observed phenotype.

**Figure 4:**
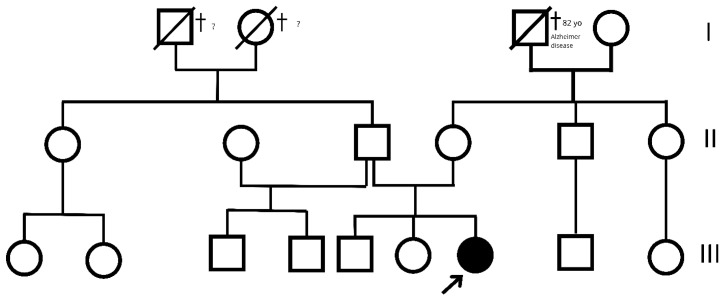
Family tree. The empty squares and circles indicate males and females, respectively. The corresponding filled-in symbols indicate the affected subjects in the family. The arrow indicates the proband. The crossed-out symbols indicate deceased subjects. Roman numerals symbolise generation. Related subjects are connected by horizontal or vertical lines, depending on the type of relationship. “†” indicates a deceased family member. “?” denotes unknown age at death or cause of death.

Familial segregation analysis performed on the mother by Sanger sequencing excluded the presence of both variants in the *PHIP* and *ANKRD17* genes. Since the father was not tested, it is not possible to conclusively determine the inheritance of the *PHIP* variant, although its absence in the mother and the negative paternal family history suggest a likely de novo origin (Figure 4).

The actual knowledge about the *PHIP*-related condition and the identified variant, together with the overlapping clinical phenotype of our patient, led us to diagnose Chung–Jansen syndrome [1,2,5]. During the genetic counselling, the clinical features and the autosomal dominant inheritance pattern of the condition together with the reproductive risks have been discussed with the patient and her mother [6]. The importance of multidisciplinary follow-up and the possibility of genetic testing for siblings, following dedicated counselling, were also explained.

From a reproductive perspective, the patient has a 50% risk of transmitting the *PHIP* variant and therefore the associated syndrome with each pregnancy, regardless of the sex of the offspring. Given the variable expressivity of the condition, the severity of the expected clinical picture in case of transmission is not predictable a priori [6]. The same risk applies to the *ANKRD17* variant and the Xp11.23 microdeletion, although their clinical significance remains uncertain.

Reproductive genetic counselling with the partner was also offered, as well as to the patient’s siblings.

### 2.3. Therapeutic Intervention

Based on the clinical and genetic findings, a multidisciplinary management pathway was then initiated. From an endocrinological perspective, therapy with 50 µg/day levothyroxine and 500 mg metformin twice daily to treat subclinical hypothyroidism and hyperinsulinism, respectively, was initiated. To address PMOS, transdermal cyclic estrogen-progestin therapy with ethinylestradiol (600 μg/week) and norelgestromin (6 mg/week) was started. The patient was taken in charge by a dietitian for the development of a hypocaloric and structured nutritional program aimed at controlling hyperphagia and achieving weight reduction. Maintenance of physical activity was also encouraged: the patient began cycling to work for approximately thirty minutes daily, reporting a beneficial effect on mood. Psychiatric follow-up was confirmed and optimised, with continuation of lithium carbonate therapy and addition of quetiapine 50 mg/day as needed for the management of acute anxiety. The patient showed optimal compliance.

### 2.4. Follow-Up and Outcomes

Six months after diagnosis, a slight weight reduction of approximately 3 kg was observed, along with an improvement in glycemic control with HOMA-IR reduced to 4.5, and improved mood stability, with a reduction in the frequency and intensity of anxiety episodes, with patient satisfaction.

## 3. Discussion

We describe the case of a young woman with Chung–Jansen syndrome diagnosed at the age of 21 years through Whole Exome Sequencing, at the conclusion of a long diagnostic journey that began in childhood to assess her psychomotor development delay and obesity. Our patient shows the typical presentation reported for the syndrome, confirming the association between *PHIP* gene variants and a phenotype characterised by intellectual disability, severe obesity early-onset, and pathognomonic facial features [1,2,8], but also offering insights into aspects less frequently explored in the existing literature.

A literature search was performed using the PubMed database and included articles published from 2016, when the syndrome was first described, to April 2026. Owing to the rarity of the disorder, studies reporting patient cohorts and individual case reports were included. We found 10 studies on patients carrying *PHIP* gene mutations associated with Chung–Jansen syndrome, for a total of 113 patients, including 55 females and 58 males.

The phenotype reported for these patients is heterogeneous, although some features are more recurrent (see Supplementary Table S1).

Nearly all described cases present dysmorphic features including large ears/earlobes, prominent eyebrows, anteverted nares and long philtrum, abnormalities of the fingers and toes; among these, the presence of fifth finger clinodactyly (ranging from 15% to 64%) [2,5,6,9,10,11,12,13] and syndactyly of the second and third toes (ranging from 15% to 30%) [2,5,6,12,13], although described in the literature, were not observed in our patient. On the other hand, we performed a comprehensive characterisation of the endocrine phenotype that is scantly investigated in the previously reported cases.

**Figure 5:**
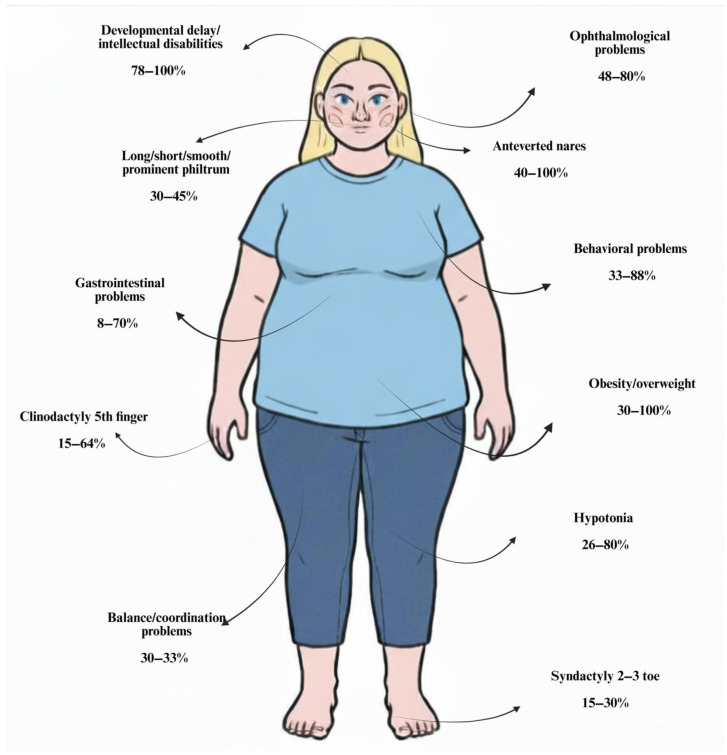
The following image summarises our patient’s characteristics: round face, upslanting palpebral fissures, prominent cheekbones, anteverted nares, a prominent chin and bilateral brachydactyly of the fourth ray. It lists the main features associated with Chung–Jansen syndrome and their reported frequency in various studies.

Our patient exhibits features such as brachydactyly (ranging from 30% to 100%) [5,9,10,11,14], upslanting palpebral fissures (ranging from 13% to 100%) [2,5,6,11] and anteverted nares (ranging from 40% to 100%) [5,6,9]. For the first two characteristics, the 100% value derives from a single-patient case report (Figure 5).

Data in the literature shows that obesity is a relatively frequent condition (on average around 70%) in patients with Chung–Jansen syndrome, in some cases described as early as childhood onset [9,12]. The study conducted by Sudnawa et al. (2024) [13] reported a lower frequency of obesity and overweight (56%) compared to previous studies: this may be explained by the examined cohort consisting mainly of paediatric patients, with obesity/overweight beginning from approximately seven years of age and gradually increasing with age. According to another study [5], the incidence of obesity/overweight in Chung–Jansen patients increases markedly during puberty; however, some patients were already affected by obesity in childhood.

Only one patient in the reported series received treatment with a GLP-1 receptor agonist (GLP-1RA), specifically liraglutide [9]. Treatment was initiated at 15 years of age and was associated with an initial favourable response, with a 6% reduction in body weight over a 5-month period. However, further studies in patients carrying PHIP gene variants are needed to determine the long-term efficacy and safety of GLP-1RA therapy in this population.

Given the involvement of PHIP gene products in the leptin–melanocortin pathway, and the hypothesis that pathogenic PHIP variants may interfere with POMC gene transcription, it could be speculated that patients with Chung–Jansen syndrome might potentially benefit from treatment with setmelanotide, a melanocortin-4 receptor (MC4R) agonist. Nevertheless, this hypothesis remains entirely speculative, as no clinical studies or evidence currently support the use of setmelanotide in this condition.

Impaired glucose tolerance with associated hyperinsulinism, autoimmune hypothyroidism, and PMOS appear to be integral components of the phenotype rather than mere comorbidities secondary to obesity. Although severe obesity can itself induce hyperinsulinism and menstrual irregularities through insulin resistance and alterations of the gonadal axis, the extent of hyperinsulinism and the presence of relatively early-onset hypothyroidism suggest a possible direct or indirect effect of *PHIP* gene dysfunction on energy metabolism and endocrine function. *PHIP* gene products are known to interact with the IGF-1 signalling pathway and with intracellular mediators of insulin signalling, playing a role in the regulation of growth and metabolism [3]. Impairment of these pathways may contribute to both obesity and the observed metabolic dysregulation [3]. Furthermore, the association with autoimmune thyroiditis, although potentially coincidental given its high prevalence in the general population, warrants attention and may represent an additional component of the phenotype to be explored in future studies. To date, the literature on Chung–Jansen syndrome has focused predominantly on neurobehavioural and dysmorphic aspects, with less attention to the endocrine–metabolic profile [2,5,6].

Cases of hypothyroidism [6,13,14], PMOS [1,13] and impaired glucose tolerance as in our patient, as well as impaired fasting glucose and gestational diabetes [9], have been reported in the literature. The onset of diabetes mellitus has been described only in one case [13]; however, it should be considered that most of the population examined in the studies is of paediatric/adolescent age, and diabetes may develop later in adulthood.

Our patient also presents with mild grade I right hydronephrosis. Pathogenic variants in the *PHIP* gene may be associated with congenital anomalies of the kidney and urinary tract. Analysis of gene expression in human and murine embryonic tissues reveals that the *PHIP* gene plays a crucial role in nephrogenesis [15]. Between 5% and 35% of patients with Chung–Jansen syndrome present with uro-renal malformations. These patients should undergo multidisciplinary clinical evaluations to promptly identify any renal defects [15].

Other features of the syndrome described in the literature include the presence of various ophthalmological issues (ranging from 48% to 80% of patients) [2,5,6,13], consistent with our patient’s refractive error, and gastroenterological problems, the most frequent being constipation (ranging from 8% to 70% of patients) [2,5,6,9,13], an aspect that our patient does not report.

Regarding the psychiatric and cognitive profile, nearly all cases described in the literature present developmental delay (ranging from 83% to 100% of patients) and intellectual disability (ranging from 78% to 100% of patients), which typically ranges from mild to moderate. Our patient presents with moderate-to-severe intellectual disability with a full-scale IQ of 38, placing her at the more severe end of the spectrum described for the syndrome [2,5,6]. This finding raises questions about the possible correlation between specific *PHIP* variants and the severity of the cognitive phenotype. The p.(Arg110Cys) pathogenic variant is located in the pleckstrin homology domain of the *PHIP* protein and may have a particularly significant impact on protein function [1,3], compromising protein–protein interactions and correct membrane anchoring, with downstream effects on gene transcription and cell signalling. However, we cannot exclude that other factors, genetic or environmental, may have modulated the expressivity of the phenotype. Indeed, the patient’s personal life experience may have influenced the psychiatric and cognitive profile of the patient, who lived for 8 years outside her family environment in a community for minors. In addition, the patient carries a maternal Xp11.23 microdeletion of approximately 5 kb, disrupting the *ZNF41* gene (OMIM Morbid gene) at a breakpoint within the gene region, as well as a heterozygous variant of uncertain significance (VUS) in *ANKRD17*. Although both findings are currently classified as VUS, a potential modifying effect on the phenotype cannot be excluded, particularly regarding the severity of intellectual disability and specific behavioural features.

The *ZNF41* gene encodes a zinc finger protein involved in transcriptional regulation and has been implicated in X-linked intellectual disability; however, the clinical relevance of small or intragenic deletions remains to be fully established. Similarly, *ANKRD17* is a candidate gene for neurodevelopmental disorders, with pathogenic variants associated with Chopra–Amiel–Gordon syndrome.

Overall, while the PHIP variant likely represents the main molecular finding underlying the clinical phenotype, the potential contribution of the ANKRD17 variant and the Xp11.23 microdeletion remains uncertain. The coexistence of these genomic alterations may contribute to phenotypic variability and raises the possibility of oligogenic effects or gene–gene interactions; however, this hypothesis remains speculative and requires confirmation through additional case reports and functional studies.

From a psychiatric perspective, the presence of anxiety, episodes of impulse disorder, and the description of elementary hallucinatory phenomena (“inner voices”) in the absence of overt psychotic disorder aligns with reports of behavioural instability and psychiatric vulnerability in Chung–Jansen syndrome [2,5].

Most studies in the literature describe the presence of behavioural problems and mood disorders in over 70% of patients [2,5,6,13]. Reported manifestations include attention-deficit/hyperactivity disorder (ADHD), attention deficits, autism spectrum disorder (ASD) traits, anxiety, mood disorders, impulsivity, aggressive behaviour, and emotional dysregulation [1,2,6]. Additional neuropsychiatric features, including sensory processing abnormalities, obsessive–compulsive behaviours, tic disorders, and other psychiatric manifestations, have been described less frequently [6]. Notably, behavioural difficulties may emerge or worsen with age and can substantially affect quality of life and functional independence [5]. These observations underscore the importance of long-term neuropsychiatric surveillance and multidisciplinary management in individuals with PHIP variants [5].

The patient’s ability to develop self-regulation strategies during her time in the community and to successfully use them to manage episodes of anger and anxiety underscores the importance of early and structured psychoeducational and cognitive–behavioural intervention. Psychiatric and psychological support should be considered an essential component of the treatment plan for patients with this syndrome.

The differential diagnosis in patients with early onset obesity is broad and complex, and its assessment needs a multidisciplinary approach including a dysmorphology genetic evaluation. Indeed, early-onset obesity may have a genetic cause.

Genetic forms of obesity can be divided into non-syndromic monogenic obesity, syndromic obesity and polygenic obesity, each characterised by specific molecular mechanisms and clinical manifestations (Supplementary Figure S1). Non-syndromic monogenic forms are generally caused by mutations in genes involved in the central regulation of appetite [16].

The leptin–melanocortin pathway represents the central regulatory axis of human energy homeostasis. In healthy individuals, leptin released by adipose tissue signals energy sufficiency to the hypothalamus, where activation of pro-opiomelanocortin (POMC) neurons promotes satiety and limits food intake while supporting appropriate energy expenditure [4,17]. PHIP has emerged as an important regulator within this network, enhancing POMC transcription and thereby contributing to the maintenance of normal body weight. In contrast, genetic defects affecting components of the leptin–melanocortin pathway disrupt satiety signalling and predispose one to severe early-onset obesity. Pathogenic variants in *LEP*, *LEPR*, *POMC* and *MC4R* genes are established causes of monogenic obesity, whereas PHIP variants appear to impair POMC expression through defective transcriptional regulation. The resulting attenuation of melanocortin signalling may contribute to hyperphagia, reduced energy balance control and excessive weight gain.

On the other hand, in syndromic forms, obesity is part of a more complex and multisystemic clinical picture, in which increased adipose mass is associated with other phenotypic manifestations such as intellectual disability, developmental abnormalities, endocrine alterations and dysmorphic features. Among the syndromes most frequently associated with obesity are Prader–Willi syndrome, Bardet–Biedl syndrome, Borjeson–Forssman–Lehmann syndrome and Cohen syndrome, rare genetic conditions in which obesity is only one element of a broader phenotype.

The identification of genetic obesity and the distinction between syndromic and non-syndromic forms have a fundamental clinical importance, as in directing the follow up, therapeutic approach and genetic counselling.

In this context, NGS confirms its role as the tool of choice for resolving these diagnoses, especially in cases with negative family history, atypical presentation, and, as in this case, a long inconclusive diagnostic journey. The diagnosis of Chung–Jansen syndrome, although delayed, allowed the conclusion of a diagnostic process lasting years, the initiation of targeted interventions, and the provision of appropriate genetic counselling to the patient and her family.

The limitations of this report are those inherent to the nature of a case report. The absence of genetic testing in the father does not allow absolute certainty regarding the de novo nature of the variant, although its absence in the mother and the negative family history make this scenario highly likely. Furthermore, the presence of additional variants of uncertain significance (Xp11.23 microdeletion and *ANKRD17* variant) introduces elements of complexity in phenotype interpretation, making it difficult to precisely establish the contribution of each variant to the overall clinical picture. Finally, the short-term follow-up does not allow evaluation of the long-term impact of the therapeutic interventions, particularly regarding weight management and psychiatric stabilisation.

## 4. Conclusions

This case report describes a young woman with Chung–Jansen syndrome due to the likely pathogenic variant c.328C>T [p.(Arg110Cys)] in the *PHIP* gene. The case confirms the association of this specific missense variant with the classic phenotype of the syndrome, characterised by the triad of intellectual disability, severe obesity, and dysmorphic features.

Of particular interest is the detailed endocrinological characterisation, which documented a picture of autoimmune hypothyroidism, severe obesity, impaired glucose tolerance with hyperinsulinism, and PMOS.

This case suggests the inclusion of comprehensive endocrine evaluations in future studies on patients with Chung–Jansen syndrome, in order to support endocrine work-up and facilitate early identification and appropriate management of potentially treatable alterations.

The case also reinforces the central role of WES in the diagnostic workup of syndromic obesity with intellectual disability, even in adulthood, especially in the presence of a long inconclusive diagnostic journey. The genetic diagnosis made it possible to end the diagnostic uncertainty, initiate targeted specialist follow-up, and provide appropriate genetic counselling.

The management of Chung–Jansen syndrome requires a multidisciplinary approach that includes a clinical geneticist, endocrinologist, nutritionist, and psychiatrist. Early diagnosis is essential to facilitate timely interventions, improve quality of life for patients and families, and prevent or manage metabolic and psychiatric complications.

In addition, a heterozygous variant of uncertain significance (VUS) in the *ANKRD17* gene was identified. Although its clinical significance remains uncertain, given the gene’s emerging role in neurodevelopmental disorders and the absence of clear genotype–phenotype correlation in this case, a potential contributory or modifying effect cannot be completely excluded. However, its role in the observed phenotype remains speculative and requires further investigation.

Further studies will be essential to delineate the mutational and phenotypic spectrum of Chung–Jansen syndrome, to clarify genotype–phenotype correlations, and to develop evidence-based recommendations for the clinical follow-up and management.

In conclusion, we report a novel endocrinological characterisation that broadens the understanding of the Chung–Jansen syndrome. Our findings emphasise the need for close collaboration between endocrinologists and other specialists, supporting a multidisciplinary approach.

**Key findings:**

- central role of WES in the diagnostic workup of syndromic obesity with intellectual disability
- importance of a detailed endocrinological characterisation
- fundamental relevance of a multidisciplinary approach

## Figures and Tables

**Table 1:** WAIS-IV Psychometric Results.

WAIS-IV Index	Score
Verbal Comprehension Index (VCI)	51
Perceptual Reasoning Index (PRI)	50
Working Memory Index (WMI)	52
Processing Speed Index (PSI)	56
Full-Scale Intelligence Quotient (FSIQ)	**38**

**Table 2:** PHIP and ANKRD17 variants.

Gene	Variant (c.xxx e p.xxx)	Exon	Zygosity	Inheritance	Frequency GnomAd	Predictive Programs	ClinVar	ACMG
PHIP NM_017934.7	c.328C>T p.Arg110Cys	5/40	HET	AD	\	AlphaMissense Deleterius. (0.971) (Moderate)	2 P, 5 LP	LP (PS2-PM2-PM5-PP2-PP5)
ANKRD17 NM_001286771.3	c.3416G>A p.Arg1139Gln	20/34	HET	AD	\	AlphaMissense Deleterius. (0.982) (Moderate)	\	VUS (PM2-PP2)

## Data Availability

The data supporting the case report are not publicly available due to patient confidentiality. No new data were generated for the narrative literature review; all information is based on previously published studies cited in the manuscript.

## Supplementary Materials

The following supporting information can be downloaded at: https://www.mdpi.com/article/10.3390/jcm15124609/s1, Table S1: clinical features of individuals with PHIP variants across studies; Supplementary Figure S1: Genetic forms of obesity.

## Abbreviations

The following abbreviations are used in this manuscript:

CHUJANS	Chung–Jansen syndrome
PHIP	Pleckstrin Homology Domain Interacting Protein
IGF-1	Insulin-like Growth Factor 1
BMI	Body Mass Index
IQ	Intelligence Quotient
PMOS	Polyendocrine Metabolic Ovarian Syndrome
NGS	Next-Generation Sequencing
Arg	Arginine
Cys	Cysteine
ACMG	American College of Medical Genetics and Genomics
ANKRD17	Ankyrin Repeat Domain 17
Array-CGH	Array-based Comparative Genomic Hybridisation
OMIM	Online Mendalian Inheritance in Man
DCAF14	DDB1- and CUL4-Associated Factor 14
NDRP	Neuronal Differentiation-Related Protein
kDa	kilodalton
IRS	Insulin Receptor Substrate
REPID	Replication initiation determinant protein
POMC	Proopiomelanocortin
WES	Whole Exome Sequencing
WHtR	Waist-to-Height Ratio
WAIS	Wechsler Adult Intelligence Scale
TSH	Thyroid Stimulating Hormone
FT3 and FT4	Free Thyroxine
HOMA-IR	Homeostatic Model Assessment for Insulin Resistance
ISCA	International Standards for Cytogenomic Arrays
OGT	Oxford Gene Technology
kB	kilobyte
ZNF41	Zinc Finger Protein 41
CNV	Copy Number Variation
HPO	Human Phenotype Ontology
AMP	Association for Molecular Pathology
PS	Strong Pathogenic
PM	Moderate Pathogenic
PP	Supporting Pathogenic
ID	Identification/Identity
gnomAD	Genome Aggregation Database
MAF	Minor Allele Frequency
Gln	Glutamine
VUS	Variant of Uncertain Significance
CAGS	Chopra–Amiel–Gordon syndrome
µg	microgram
mg	milligram
kg	kilogram
GLP1-RA	Glucagon-Like Peptide-1 Receptor Agonists
MC4R	Melanocortin 4 Receptor
LEP	Leptin
LEPR	Leptin Receptor
PCSK1	Proprotein Convertase Subtilisin/Kexin type 1
