# Supplementary material for: Chung–Jansen Syndrome in a Young Woman with a PHIP Variant: Severe Obesity, Intellectual Disability, and Endocrine Abnormalities
Source: J Clin Med. 2026 Jun 13;15(12):4609. doi: 10.3390/jcm15124609 (PMC13301996; doi:10.3390/jcm15124609)
Supplement: Supplementary file 1 [file jcm-15-04609-s001.zip › jcm-4357938-supplementary.pdf]

Supplementary Table S1: clinical features of individuals with PHIP variants across studies

	**Our patient (n=1)**	**Loid et al. 2026 [9] (n=3)**	**Khalili et al. 2025 [12] (n=1)**	**Sudnawa et al. 2024 [13] (n=47)**	**Pascolini et al. 2024 [10] (n=1)**	**Kampmeier et al. 2023 [5] (n=23)**	**Conti et al. 2023 [14] (n=2)**	**Kaur et al. 2021 [11] (n=1)**	**Craddock et al. 2019 [6] (n=10)**	**Jansen et al. 2018 [2] (n=23)**	**Webster et al. 2016 [1] (n=2)**
Age	21 years	5 and 15 years, mother	7 years	~ 11 years (4 months-44 years)	9 years	~ 13 years (5 years-54 years)	4 years (P1), mother (P2)	7 years	~ 9 years (16 months-15 years)	~ 14 years (5 years-52 years)	8, 14 years
Gender	F	M, M, F	M	F (51%), M (49%)	M	F (43.5%), M (56.5%)	M, F	M	F (50%), M (50%)	F (52%), M (48%)	F
Prenatal issues									+ (50%)		-
Neonatal issues				+ (50%)			+ (50%) Neonatal distress and needed resuscitation with intubation and cardiac massage		Hypotonia (40%)		-
Feeding difficulties in neonatal period	-			+ (45%)		+ (18%)			+ (70%)	+ (26%)	
Neonatal hyperbilirubinemia	+			+ (38%)		+ (22%)					
Round face	+							+			+ (50%)
**Dysmorphic features**											
Brachydactyly	+	+ (33%)			+	+ (30%)	+ (50%)	+			
Clinodactyly 5th finger		+ (33%)	+	+ (15%)	+	+ (30%)	+ (5th finger) + (2nd finger)	+	+ (50%) (40% 5th finger)	+ (64%)	
Syndactyly 2/3 toe			+ (semi-syndactyly)	+ (15%)	-	+ (26%)			+ (20%)	+ (30%)	
Tapering fingers					-	+ (43%)		-		+ (76%)	
Upturned/short nose		+ (100%)			-	+ (23%)	+ (100%)	+	+ (20%)	+ (68%)	+ (50%)
Thick alae nasi					-					+ (68%)	
High forehead		+ (33%)			-			-	+ (70%)	+ (67%)	
Large/fleshy earlobes					-	+ (61%)		+	+ (20%)	+ (64%)	+ (100%)
Thick helices					-			+	+ (30%)		
Thick earlobes		+ (33%)			-			+	+ (30%)		
Anormal eyebrows		+ (33%)			-	+ (61%)	+ (100%)	-		+ (59%)	
Prominent cheeckbones	+				+						
Anterverted nares	+	+ (100%)			-	+ (52%)			+ (40%)		
Broad nasal tip					+	+ (18%)			+ (20%)		
Thin/full lips					+		+ (100%)	-	+ (60%)	+ (36%)	
Up-turned upper lip					-						+ (50%)
Long/short/smooth/prominent philtrum	+				-	+ (30%)	+ (100%)	+	+ (40%)	+ (45%)	+ (50%)
Upslanting palpebral fissures/almond-shaped eyes	+				-	+ (13%)		+	+ (50%)	+ (59%)	
Long eyelashes							+ (100%)				
Synophrys					-		+ (100%)	-	+ (30%)	+ (59%)	
Deep set eyes					-	+ (17%)					+ (50%)
Hypertelorism									+ (30%)		
Epicanthus					-			+	+ (10%)		
**Obesity/overweight**	+	+ (100%, already in childhood)	+ (since 18 months)	+ (56%)	-	+ (70%)		+	+ (30%)	+ (74%)	+ (100%)
Insulin resistance	+										+ (50%)
**Developmental delay**	+	+ (100%)	+	+ (85%)	+	+ (96%)	+ (100%)	+	+ (100%)	+ (83%)	+ (100%)
**Intellectual disabilities**	+	+ (100%)	+	+ (85%)	+	+ (91%)	+ (100%)	+	+ (100%)	+ (78%)	+ (100%)
Age at sitting	12 months								~ 11.3 months		6, 9 months

Age at walking	24-36 months	Gross motor delay (33%)	30 months					24 months	~ 23.8 months		14, 18 months
Age at talking	24 months first words<br>4-5 years complex sentences		4 years single words<br>6 years complete sentences					12 months	~ 30 months		16, 18 months (first words), sentences at 4 years
Full-scale IQ	38						48 (P2)		~ 64 (n=4)		60
Sleeping difficulties				+ (43%)		+ (26%)				+ (18%)	
Anxiety or depression	+			+ (47%)							+ (100%)
Brain MRI/CT anormalities							Thinned corpus callosum, malrotation of the hippocampi and a thinning of the olfactory bulbs (P1)	-			
Seizure	-		+	+ (6%)		+ (17%)			+ (20%)	+ (4.3%)	
Fatigue					-			+	+ (70%)	+ (56%)	
Behavioral problems and mood disorder	+	+ (33%)	+	+ (70%)	-	+ (87%)		+	+ (88%)	+ (78%)	+ (50%)
Hypotonia	+		+	+ (79%)		+ (35%)		+	+ (80%)	+ (26%)	+ (100%)
Balance/coordination problems		+ (33%)		+ (30%)		+ (30%)					+ (100%)
Hyperactivity			+	+ (51%)							
Gastrointestinal problems		Constipation (33%)		Constipation (49%)		Constipation (35%)	Esophageal atresia with fistula (50%)		Constipation (70%)<br>GERD (40%)	Constipation (8%)	Constipation, diarrhea (50%)
Ophthalmological problems	+			+ (66%)	+	+ (48%)		+	+ (80%)	+ (65%)	+ (50%)
Morphological kidney anomalies	+			+ (8%)			Horseshoe kidneys with differentiated parenchyma (50%)	+ (small right kidney, decreased corticomedullary differentiation)			
Undescended testis (crypthorchidism)				+ (39%)		+ (26%)			+ (20%)	+ (27%)	
Endocrine problems	PMOS<br>Hypothyroidism in autoimmune thyroid disease	Gestational diabetes (mother)<br>Impaired glucose tolerance/prediabetes (66%)		PMOS (8%)<br>Late puberty (6%)<br>Precocious puberty (4%)<br>Diabetes mellitus (4%)<br>Hypothyroidism (2%)<br>Ectopic pituitary (2%)			Insulin-dependent diabetes (50%),<br>Hypothyroidism (50%)		Hypothyroidism (10%)<br>Delayed menarche (20%)		PMOS (50%)
Skin problems								-	+ (40% CAL)		
Other notes							Biventricular nonobstructive cardiac hypertrophy (50%),<br>Hypertrophic cardiomiopathy of the left ventricle (50%)				

Supplementary Figure S1

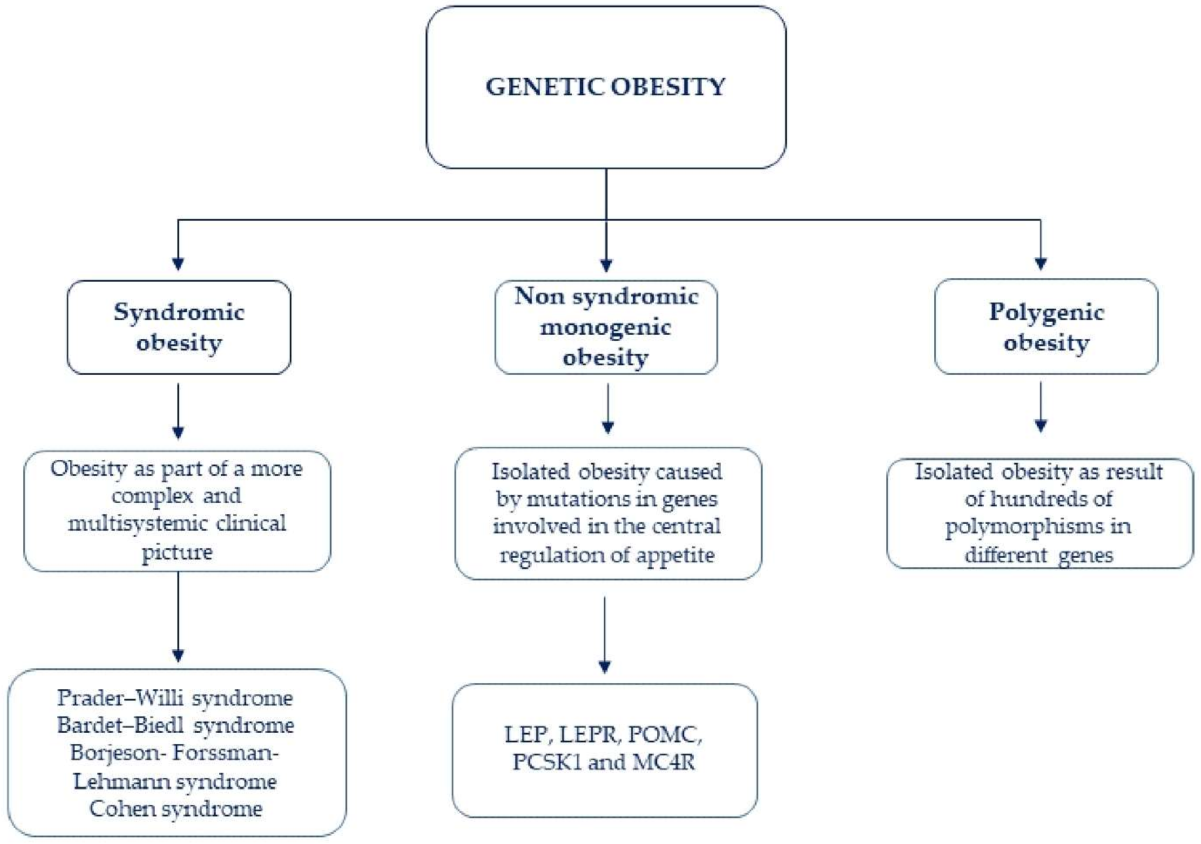

Short Biography of the first Author

Francesco Donno is a final-year medical resident specializing in Endocrinology and Metabolic Diseases at the University of Ferrara. He earned his Medical Degree with honors (110/110 cum laude) from the University of Bologna during the 2019/2020 academic year and he is currently registered with the Medical Association of Lecce. His clinical experience includes working as a Medical Physician in the Emergency Department at Vito Fazzi Hospital in Lecce. During his residency, he completed the Thyroid Masterclass organized by Collegium POEMA in November 2025. Dr. Donno has actively contributed to the academic field as a thesis co-supervisor and speaker at various seminars and conferences. He has presented several posters at national congresses and contributed scientific articles to the "Novità" section of *L'Endocrinologo* and to *AME News*. He is an active member of both the Italian Society of Endocrinology (SIE) and the Association of Medical Endocrinologists (AME). Previously, he was listed in the National Register of Excellence after graduating from the "F. Capece" Classical Lyceum with maximum honors.
